# Supplementary material for: Study protocol for connective tissue disease-associated interstitial lung disease trial (TEL-CTD-ILD): A randomized controlled trial of a home-based telemonitoring of treatment effects
Source: PLoS One. 2022 Dec 27;17(12):e0278601. doi: 10.1371/journal.pone.0278601 (PMC9794074; doi:10.1371/journal.pone.0278601)
Supplement: S1 File — (PDF) [file pone.0278601.s002.pdf]

Łódź, 03/01/2019

Dr hab. med. Prof. UM Wojciech Piotrowski

Department of Pneumonology and Allergology,

Department of Internal Medicine

University Clinical Hospital No. 1 N. Barlicki in Łódź

Tel .: 42 678-78-05

Chairman of the Bioethics Committee

at the Medical University of Lodz

Application for an opinion on human medical research

<u>Principal Investigator and Contractor:</u>

1. **Principal researcher:** Wojciech Piotrowski, MD, prof. Medical University in Łódź (specialist in internal diseases, specialist in lung diseases), Department of Pneumology and Allergology, Medical University of Łódź

Co-researchers:

2. Maria Mozga (MD, no specialization), Department of Pneumology and Allergology, Medical University of Łódź

3. Sylwia Małysiak-Szpond (MA in Biology), Roche Polska

Unit name and address:

Department of Pneumonology and Allergology, Department of Internal Medicine

Project title (in Polish):

Monitoring the natural history and treatment effects of selected interstitial lung diseases in the course of connective tissue diseases (CTD-ILD)

Defining the type of research experiment:

Clinical research project.

Study description:

Introduction

Interstitial lesions are a common organ manifestation of connective tissue diseases such as scleroderma, rheumatoid arthritis (RA), Sjögren's syndrome, systemic lupus or dermatomyositis [1]. The most common radiological and histopathological patterns are nonspecific interstitial pneumonia (NSIP), organizing pneumonia (OP), common interstitial pneumonia (UIP), and lymphocytic pneumonia (LIP). In most cases, these changes are inflammatory, with a variable proportion of interstitial fibrosis. In the high-resolution computed tomography (HRCT) examination, one can distinguish features clearly supporting fibrosis (honeycomb, pull distortions), other radiological features, such as reticular lesions or the milky glass, may represent both areas of early fibrosis and chronic interstitial inflammation [2]. In everyday clinical practice, after the diagnosis is made on the basis of a careful history, clinical data, HRCT picture, and sometimes lung biopsy, empirical treatment is initiated, usually consisting of a glucocorticoid and an immunosuppressive drug. However, it is difficult to predict the response to the treatment on the basis of clinical and radiological features. Its effectiveness is assessed on the basis of functional tests and tomography performed usually after 3 months of therapy. In the absence of a response to treatment, the patient is exposed to, sometimes serious, side effects associated with taking glucocorticosteroids or immunosuppression [3].

The study is designed to assess the possible benefits of telemedicine in monitoring changes in functional and vital signs, symptoms and assessing quality of life in response to treatment. We assume that the introduction of telemedicine for treatment monitoring will allow for earlier decisions regarding the continuation, dose change or discontinuation of therapy, and may also improve the quality of life of patients. It will also allow you to assess the dynamics of the recovery of changes (different patterns of response to treatment). Additionally, predictors of failure to respond to treatment will be assessed (clinical diagnosis, radiological, functional, clinical features, biomarkers).

Study group and methods

The study will include patients diagnosed with connective tissue disease- interstitial lung disease (CTD-ILD), who, on the basis of clinical and radiological features, show signs of a potentially reversible nature of the lesions (corresponding to the NSIP pattern) [1]. Each patient will undergo functional tests (spirometry, diffusion, body plethysmography, 6-minute walk test). At the time of qualification for the study, serum will be collected in order to determine the biomarkers of fibrosis (KL-6, MMP-7, SPD) [4-6]. The initial assessment of the severity of symptoms and quality of life will consist of completing the cough assessment questionnaires (Leicester cough questionnaire), dyspnea (mMRC scale, Borg scale), fatigue (FAS scale), quality of life (St. George's Hospital questionnaire, K-BILD, SF- 36).

Patients diagnosed with CTD-ILD (according to the criteria as above) will be assigned to the study group (telemonitoring, n = 30) and the control group (traditional assessment, n = 30). The division into groups will be carried out by drawing lots. Patients from the telemonitoring group, after initial training by staff, will conduct independent daily (1x daily in the morning) measurements of spirometry (FVC), percutaneous pulse oximetry, heart rate, blood pressure, activity (accelerometry) and the assessment of the severity of cough and dyspnea in 5 a two-point Likert scale [7]. Telemonitoring will start approximately 10-14 days prior to treatment initiation and will continue for 3 months of treatment. All patients (test and control groups) will receive treatment with Encorton at an initial dose of 0.5 mg/kg (prednisone) with a reduction of 5 mg every 4 weeks and azathioprine 2 mg/kg (maximum 150 mg daily) or another

immunosuppressant (mycophenolate mofetil, cyclophosphamide, cyclosporine), depending on the clinical situation. Treatment will be in accordance with the current standards of treatment and the Summary of Product Characteristics of the selected preparation. The final decision on the choice of therapy will be made by a specialist in lung diseases and will be individualized, in line with current medical knowledge. Additional questionnaires will also be used to assess the tolerance of the treatment and the occurrence of side effects. During the 3-month follow-up period, visits to the center will take place at monthly intervals. In the event of deterioration of monitored parameters below the set thresholds or failure to register them by the patient, team members will be required to contact by phone within the next 24 hours. In case of intolerance to treatment or deterioration of monitored parameters, patients will be assessed at additional time points. All patients, regardless of their allocation to the group, will remain under the care of the Pulmonology Clinic at USK No. 1 after the end of the 3-month follow-up and will be examined during regular visits every 3 months, at least until the end of the 12-month follow-up period.

Based on the results obtained, it will be possible to analyze the regression patterns of changes: progression, no response, slow improvement, fast improvement, variable response. At subsequent visits, the possibility of modifying the treatment will be assessed, e.g. drug dose reduction, treatment change, discontinuation, etc.

Aims of the work:

1. Characterization of treatment response patterns;
2. Assessment of clinical predictors of poor response to treatment;
3. To investigate the impact of telemonitoring on the quality of life (QoL) of patients with CTD-ILD with an inflammatory component.
4. Cost-effectiveness evaluation of CTD-ILD treatment telemonitoring

Inclusion criteria:

1. Newly diagnosed interstitial lung disease with a small component of fibrous changes (changes in the lungs in the course of connective tissue disease with the NSIP pattern);
2. Indications for therapy with systemic glucocorticosteroid +/- immunosuppressive drug;
3. 18 years of age or older;
4. Expressing a written consent to participate in the study;
5. Effective contraception
6. The result of the Mini Mental test ensuring the possibility of efficient operation of monitoring devices
7. Completed training in the use of telemedicine equipment

Exclusion criteria:

1. Idiopathic pulmonary fibrosis (IPF) or fibrosing NSIP
2. Pattern of certain or probable common pneumonia (UIP) organizing pneumonia (OP), lymphocytic pneumonia (LIP) on HRCT study
3. Contraindications to treatment with glucocorticosteroids and immunosuppressants (azathioprine or mycophenolate mofetil or cyclophosphamide or cyclosporine)
4. Pregnancy and breastfeeding

Literature

1. Travis WD, Costabel U, Hansell DM, King TE, Lynch DA, Nicholson AG, et al. An official American Thoracic Society / European Respiratory Society statement: Update of the international multidisciplinary classification of the idiopathic interstitial pneumonias. Am J Respir Crit Care Med. 2013 Sep 15; 188 (6): 733-48.

2. Khanna D, Mittoo S, Aggarwal R, Proudman SM, Dalbeth N, Matteson EL, et al. Connective Tissue Disease-associated Interstitial Lung Diseases (CTD-ILD) - Report from OMERACT CTD-ILD Working Group. J Rheumatol. 2015 Nov; 42 (11): 2168–71.

3. Lee JY, Jin S-M, Lee BJ, Chung DH, Jang B-G, Park HS, et al. Treatment response and long term follow-up results of nonspecific interstitial pneumonia. J Korean Med Sci. 2012 Jun; 27 (6): 661–7.

4. Bauer Y, White ES, de Bernard S, Cornelisse P, Leconte I, Morganti A, et al. MMP-7 is a predictive biomarker of disease progression in patients with idiopathic pulmonary fibrosis. ERJ Open Res. 2017 Jan; 3 (1).

5. Wakamatsu K, Nagata N, Kumazoe H, Oda K, Ishimoto H, Yoshimi M, et al. Prognostic value of serial serum KL-6 measurements in patients with idiopathic pulmonary fibrosis. Respir Investig. 2017 Jan; 55 (1): 16-23.

6. Ikeda K, Shiratori M, Chiba H, Nishikiori H, Yokoo K, Saito A, et al. Serum surfactant protein D predicts the outcome of patients with idiopathic pulmonary fibrosis treated with pirfenidone. Respir Med. 2017; 131: 184–91.

7. Miłkowska-Dymanowska J, Białas AJ, Obrebski W, Górski P, Piotrowski WJ. A pilot study of daily telemonitoring to predict acute exacerbation in chronic obstructive pulmonary disease. Int J Med Inf. August 2018; 116: 46–51.

Information on safety conditions

Insurance on general terms

Attachments:

1. Study protocol. 2. Information for the Patient. 3. Patient's Informed Consent Form. 4. Principal Investigator CV (signed). 5. List of centers participating in the study. 6. Consent of the Director of the Hospital
