# Supplementary material for: Study protocol for connective tissue disease-associated interstitial lung disease trial (TEL-CTD-ILD): A randomized controlled trial of a home-based telemonitoring of treatment effects
Source: PLoS One. 2022 Dec 27;17(12):e0278601. doi: 10.1371/journal.pone.0278601 (PMC9794074; doi:10.1371/journal.pone.0278601)
Supplement: Attachment — Submitted filename: Response to Reviewers.docx [file pone.0278601.s003.docx]

PONE-D-21-39284

Study protocol for Connective Tissue Disease-associated Interstitial Lung Disease Trial (TEL-CTD-ILD): a randomized controlled trial of a home-based telemonitoring of treatment effects

PLOS ONE

Response to Reviewers

We would like to thank the Reviewers for their input and valuable comments. The answers follow the questions and comments formulated by Editor/Reviewer:

Journal Requirements:

When submitting your revision, we need you to address these additional requirements.

1. Please ensure that your manuscript meets PLOS ONE's style requirements, including those for file naming. The PLOS ONE style templates can be found at
   https://journals.plos.org/plosone/s/file?id=wjVg/PLOSOne_formatting_sample_main_body.pdf and 
   <https://journals.plos.org/plosone/s/file?id=ba62/PLOSOne_formatting_sample_title_authors_affiliations.pdf>

**Answer: done according to instruction.**

2. We note that the grant information you provided in the ‘Funding Information’ and ‘Financial Disclosure’ sections do not match.

When you resubmit, please ensure that you provide the correct grant numbers for the awards you received for your study in the ‘Funding Information’ section.

**Answer: Grant number and name of funding institution in Financial Disclosure is correct. Potentially confusing information on funding was deleted form the Acknowledgements.**

3. Thank you for stating the following in the Acknowledgments Section of your manuscript: "The authors would like to thank Polish Ministry of Science and Higher Education for funding support; the study participants and staff at Department of Pneumology of the Medical University of Lodz for their involvement of this trial."

We note that you have provided funding information that is not currently declared in your Funding Statement. However, funding information should not appear in the Acknowledgments section or other areas of your manuscript. We will only publish funding information present in the Funding Statement section of the online submission form. 
Please remove any funding-related text from the manuscript and let us know how you would like to update your Funding Statement. Currently, your Funding Statement reads as follows: "The study is funded by the Polish Ministry of Science and Higher Education (0047/DW/2018)."

Please include your amended statements within your cover letter; we will change the online submission form on your behalf.

**Answer: We deleted funding information from the Acknowledgments.**

4. In your Data Availability statement, you have not specified where the minimal data set underlying the results described in your manuscript can be found. PLOS defines a study's minimal data set as the underlying data used to reach the conclusions drawn in the manuscript and any additional data required to replicate the reported study findings in their entirety. All PLOS journals require that the minimal data set be made fully available. For more information about our data policy, please see http://journals.plos.org/plosone/s/data-availability.

Upon re-submitting your revised manuscript, please upload your study’s minimal underlying data set as either Supporting Information files or to a stable, public repository and include the relevant URLs, DOIs, or accession numbers within your revised cover letter. For a list of acceptable repositories, please see http://journals.plos.org/plosone/s/data-availability#loc-recommended-repositories. Any potentially identifying patient information must be fully anonymized.

Important: If there are ethical or legal restrictions to sharing your data publicly, please explain these restrictions in detail. Please see our guidelines for more information on what we consider unacceptable restrictions to publicly sharing data: http://journals.plos.org/plosone/s/data-availability#loc-unacceptable-data-access-restrictions. Note that it is not acceptable for the authors to be the sole named individuals responsible for ensuring data access.

We will update your Data Availability statement to reflect the information you provide in your cover letter.

**Answer: As mentioned in the Material and methods section “Data collection for the study will be completed by June 2023”. The article is a study protocol description; therefore, data are not available at this stage. The answer to the question should be - data availability policy is not applicable to this article.**

5. We note that you have stated that you will provide repository information for your data at acceptance. Should your manuscript be accepted for publication, we will hold it until you provide the relevant accession numbers or DOIs necessary to access your data. If you wish to make changes to your Data Availability statement, please describe these changes in your cover letter and we will update your Data Availability statement to reflect the information you provide.

**Answer: the repository information for our data is not yet available. This article does not report data**

6. Please review your reference list to ensure that it is complete and correct. If you have cited papers that have been retracted, please include the rationale for doing so in the manuscript text, or remove these references and replace them with relevant current references. Any changes to the reference list should be mentioned in the rebuttal letter that accompanies your revised manuscript. If you need to cite a retracted article, indicate the article’s retracted status in the References list and also include a citation and full reference for the retraction notice.

**Answer: not applicable. Reference list is complete and correct.**


Reviewers' comments:

Reviewer's Responses to Questions

**Comments to the Author**

1. Does the manuscript provide a valid rationale for the proposed study, with clearly identified and justified research questions?

The research question outlined is expected to address a valid academic problem or topic and contribute to the base of knowledge in the field.

Reviewer #1: Yes

Reviewer #2: Yes

**Answer: thank you, we appreciate your opinion**

2. Is the protocol technically sound and planned in a manner that will lead to a meaningful outcome and allow testing the stated hypotheses?

The manuscript should describe the methods in sufficient detail to prevent undisclosed flexibility in the experimental procedure or analysis pipeline, including sufficient outcome-neutral conditions (e.g. necessary controls, absence of floor or ceiling effects) to test the proposed hypotheses and a statistical power analysis where applicable. As there may be aspects of the methodology and analysis which can only be refined once the work is undertaken, authors should outline potential assumptions and explicitly describe what aspects of the proposed analyses, if any, are exploratory.

Reviewer #1: Yes

Reviewer #2: Yes

**Answer: thank you, we appreciate your opinion**

3. Is the methodology feasible and described in sufficient detail to allow the work to be replicable?


Descriptions of methods and materials in the protocol should be reported in sufficient detail for another researcher to reproduce all experiments and analyses. The protocol should describe the appropriate controls, sample size calculations, and replication needed to ensure that the data are robust and reproducible.

Reviewer #1: Yes

Reviewer #2: Yes

**Answer: thank you, we appreciate your opinion**

4. Have the authors described where all data underlying the findings will be made available when the study is complete?

The [PLOS Data policy](https://journals.plos.org/plosone/s/materials-and-software-sharing) requires authors to make all data underlying the findings described in their manuscript fully available without restriction, with rare exception, at the time of publication. The data should be provided as part of the manuscript or its supporting information, or deposited to a public repository. For example, in addition to summary statistics, the data points behind means, medians and variance measures should be available. If there are restrictions on publicly sharing data—e.g. participant privacy or use of data from a third party—those must be specified.

Reviewer #1: Yes

Reviewer #2: Yes

5. Is the manuscript presented in an intelligible fashion and written in standard English?

PLOS ONE does not copyedit accepted manuscripts, so the language in submitted articles must be clear, correct, and unambiguous. Any typographical or grammatical errors should be corrected at revision, so please note any specific errors here.

Reviewer #1: Yes

Reviewer #2: Yes

**Answer: Thank you, we appreciate your opinion**

6. Review Comments to the Author

**The Authors would like to thank the Reviewers for thorough assessment and valuable comments. We believe, corrections applied in response to these comments allowed to improve the legibility and quality of our work.**

Reviewer #1: 1. The sample size calculation does not seem correct. The margin 0.05 is tool small in relative to SD15.8. N 26 should not have enough power.

**Answer: The calculation was done considering quality of life measured by SGRQ, as the primary endpoint of the study. We are aware, that achieving other endpoints may deserve higher numbers of patients. Therefore, first data report will be treated as preliminary, and further studies will be planned, potentially multicenter, to achieve other endpoints.**

2. Linear mixed model can only handle missing at random.

**Answer: random missing data are expected in this study**

Reviewer #2: The authors have created a clear protocol to analyze telemonitoring in patients with CTD-ILD starting treatment compared to standard of care. They have stated clear objectives such as the effect of telemonitoring on quality of life, which in a prior asthma study showed a small improvement, and other punctual secondary objectives which are also important to consider such as cost-effectiveness of the approach. Although not the focus of this study, the results may pave the way for future use of telemonitoring to more closely evaluate treatment response (home vs office based spirometry results and correlation with HRCT chest at follow up).
The protocol is presented clearly, the methods are well described. I suggest, if not already considered, that the treatment each patient receives should be specified and also consider including perceived side effects from treatment as part of patient questionnaires.

**Answer: Thank you for your opinion. We plan to describe details of treatment, such as steroid daily dose, type of immunosuppressive drug etc. We also included a questionnaire assessing potential side effects of drugs.**
